# An integrative meta-analysis of SARS-CoV-2 RNA–protein interactomes identifies conserved host factors shared with other RNA viruses

**DOI:** 10.1093/bfgp/elag001

**Published:** 2026-05-18

**Authors:** Kuerbannisha Amahong, Yuhong Liu, Zheng Zhang, Lin Tao, Aishe A Sarshad, Feng Zhu

**Affiliations:** College of Pharmaceutical Sciences, The Second Affiliated Hospital, Zhejiang University School of Medicine, Zhejiang University, Hangzhou 310058, China; Department of Medical Biochemistry and Cell Biology, Institute of Biomedicine, University of Gothenburg, SE-40530 Gothenburg, Sweden; Wallenberg Centre for Molecular and Translational Medicine, University of Gothenburg, SE-40530 Gothenburg, Sweden; Key Laboratory of Elemene Class Anti-cancer Chinese Medicines, School of Pharmacy, Hangzhou Normal University, Hangzhou 311121, China; Key Laboratory of Elemene Class Anti-cancer Chinese Medicines, School of Pharmacy, Hangzhou Normal University, Hangzhou 311121, China; Key Laboratory of Elemene Class Anti-cancer Chinese Medicines, School of Pharmacy, Hangzhou Normal University, Hangzhou 311121, China; Department of Medical Biochemistry and Cell Biology, Institute of Biomedicine, University of Gothenburg, SE-40530 Gothenburg, Sweden; Wallenberg Centre for Molecular and Translational Medicine, University of Gothenburg, SE-40530 Gothenburg, Sweden; College of Pharmaceutical Sciences, The Second Affiliated Hospital, Zhejiang University School of Medicine, Zhejiang University, Hangzhou 310058, China

**Keywords:** RNA virus, virus-host interactions, immune evasion, virus replication, drug repurposing

## Abstract

RNA viruses cause substantial global disease burden and depend on host RNA-binding proteins and translation machinery. However, it remains unclear which host factors are robustly engaged across independent Severe Acute Respiratory Syndrome Coronavirus 2 (SARS-CoV-2) RNA interactome studies and to what extent these factors are shared with other RNA viruses. Here, we perform an integrative meta-analysis of eight published SARS-CoV-2 RNA–protein interactomes and compare them with corresponding Influenza A virus, Zika virus, and Dengue virus datasets to define conserved host networks and prioritize candidate host-directed antiviral targets. By integrating multiple datasets and applying ClusterProfiler together with curated pathway resources (KEGG, Reactome, WikiPathways, and Gene Ontology), we systematically characterize the functional landscape of SARS-CoV-2 RNA–protein interactions. The consensus SARS-CoV-2 interactome is enriched for mRNA processing, translation, RNA surveillance and innate immune functions. Cross-viral comparison identifies 275 host proteins shared across all four RNA viruses, forming interconnected modules that include key translation factors (EEF1A1, EIF4A1, EIF3H) and RNA-binding proteins (Nucleolin, ILF3). Drug–target annotation prioritizes 21 proteins with 35 approved or investigational modulators for host-directed antiviral repurposing. Together, these findings generate a consensus map of conserved host dependencies and highlight prioritized targets for future mechanistic and translational studies.

**Research Highlights**

- Integrated SARS-CoV-2 datasets and compared with, Influenza A virus, Zika virus, Dengue virus.
- Identified 275 host proteins shared across these four pathogens.
- Conserved proteins were enriched in translation, RNA processing, and innate immune pathways.
- Prioritized 21 host targets and 35 drugs for antiviral repurposing.

## Introduction

The emergence of RNA viruses has led to severe global health crises, ranging from the widespread pandemics caused by SARS-CoV-2 and Influenza A Virus (IAV) to the significant epidemics associated with Zika Virus (ZIKV) and Dengue Virus (DENV). To establish infection and cause disease, these pathogens must effectively manipulate host cellular mechanisms [1, 2]. Therefore, understanding the specific viral-host interactions that drive these processes is essential for elucidating pathogenic mechanisms and developing effective therapeutic solutions [3, 4].

The life cycle of RNA viruses relies heavily on host cell molecules to compensate for their limited coding capacity. In response, host innate immune mechanisms have evolved to detect viral RNAs and replication intermediates, triggering antiviral defenses [5, 6]. Specifically, the host system recognizes unconventional molecular signatures such as 5′ triphosphates and double-stranded RNA to suppress viral gene expression [7–9]. Key sensors, including RIG-I and MDA5 [10] and TLR3 [11], detect these patterns and initiate signaling cascades that lead to the production of type I interferons and the establishment of an antiviral state [12]. However, RNA viruses have developed strategies to evade this detection; for instance, SARS-CoV-2 hijacks the host protein LARP1 to enhance viral replication [13], while DENV interacts with the splicing factor RBM10 to deregulate host innate immune responses [8].

In recent years, virus-RNA-protein interaction genomics have revealed how RNA viruses manipulate host signaling pathways—such as inflammation, oxidative stress, apoptosis, and autophagy—and how these interactions can be targeted therapeutically [13, 14]. For instance, drug-induced inhibition of SARS-CoV-2 RNA interactome targets, including PPIA [15], HSP90 and IGF2BP1 [16, 17] has been shown to significantly reduce viral replication. These interactions exhibit a dual role: while some host RBPs recognize viral RNA to trigger interferon responses [18], others are ‘hijacked’ to promote viral stability, such as the recruitment of ANGEL2 and La autoantigen by SARS-CoV-2 [16, 17]. Similarly, IAV, HCV, and DENV manipulate inflammatory and metabolic pathways—including DPP4-mediated cytokine release [15], NF-κB signaling, and redox states [19], while SARS-CoV-2 interacts with Bcl-2 family proteins to inhibit apoptosis [20]. Collectively, the inhibition of these targets confirms that the RNA–protein interactome provides a valuable resource for the development of antiviral therapies.

**Figure 1:**
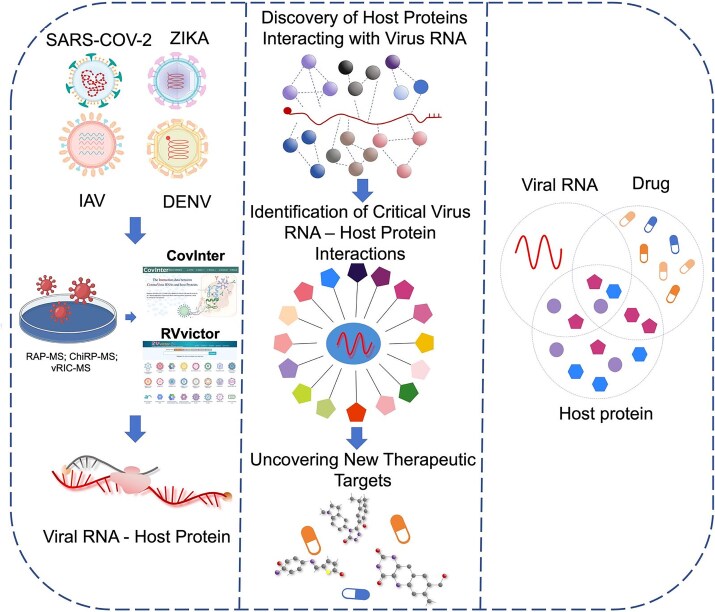
Overview of systematic analysis of viral RNA and host protein interaction data. Schematic workflow showing how published RNA capture–mass spectrometry datasets (*e.g.* RAP-MS, ChIRP-MS, vRIC-MS) reporting host proteins bound to the RNA genomes of SARS-CoV-2, IAV, ZIKV, and DENV were collected from the literature and curated in our CovInter and Rvvictor databases (left). These datasets were integrated to construct a consensus SARS-CoV-2 RNA interactome and to identify host proteins shared across all four viruses, followed by functional enrichment and network analyses (middle). Conserved host factors were finally annotated with known drug–target information to prioritize candidate targets for host-directed antiviral therapy (right).

In this study, we integrated viral RNA–host protein interaction datasets across multiple RNA viruses. Individual SARS-CoV-2 RNA–protein interactome studies use different cell types and capture methods, so any single dataset may contain method- or context-specific interactions. We therefore hypothesized that a consensus SARS-CoV-2 RNA interactome, combined with cross-viral comparison, would reveal conserved host factors that are more robust biologically and promising as candidate therapeutic targets. To test this, we performed an integrative meta-analysis of eight published SARS-CoV-2 RNA–protein interactomes and analyzed them with ClusterProfiler and curated pathway resources (KEGG, Reactome, WikiPathways, and Gene Ontology [GO]), followed by protein–protein interaction network analysis. We then compared the resulting host networks with RNA interactome datasets from IAV, ZIKV, and DENV to identify shared host modules and annotated these conserved factors with drug–target information to prioritize candidates for host-directed antiviral repurposing (Fig. 1). Together, these findings improve our understanding of how hosts respond to RNA virus infection and generate a prioritized set of candidate host-directed targets for future experimental evaluation in SARS-CoV-2 and related viruses.

## Materials and methods

### Extraction of host proteins interacting with the SARS-CoV-2 RNA

To build a robust consensus of the SARS-CoV-2 RNA interactome, we first queried CovInter, our curated database of virus RNA–host protein interactions, which archives 10,180 interaction records between 310 coronavirus RNA strains and 1281 host proteins [21]. From this resource, we applied strict inclusion and exclusion criteria to select high-confidence SARS-CoV-2 datasets. Specifically, datasets were included if they (i) employed experimentally validated RNA pull-down methods to ensure specific retrieval of RNA-binding proteins, (ii) targeted the full-length SARS-CoV-2 RNA genome rather than isolated specific regions, and (iii) provided sufficient raw RNA—protein interaction data for downstream analysis. Conversely, we excluded datasets that relied on computational or predictive methods rather than experimental validation (*e.g.* deep-learning models such as Pysster/DeepRiPe, or Position Weight Matrix predictions from the ATtRACT database), datasets targeting only specific non-coding RNA regions, and compound or redundant reference entries. After applying these criteria, eight independent datasets satisfied all inclusion requirements and were retained for analysis (Table 1). These eight studies encompass seven SARS-CoV-2 strains (hCoV-19/England/02/2020, hCoV-19/France/IDF-220-95/2020, hCoV-19/South Korea/KCDC03/2020, a clinical isolate from Germany, hCoV-19/IPBCAMS-YL01/2020, hCoV-19/Wuhan-Hu-1/2019, and hCoV-19/USA/WA1/2020) from six countries and a range of experimental techniques, including RNA antisense purification coupled with mass spectrometry (RAP-MS), Label-Free Quantification, Liquid Chromatography with Tandem Mass Spectrometry (LC–MS/MS), Tandem Mass Tag labeling, RNA purification and chromatin isolation techniques combined with mass spectrometry (ChIRP-M/S), and RNA-protein interaction detection assays (RaPID), maximizing methodological and geographical diversity to filter out method-specific artifacts and define a robust consensus interactome.

**Table 1:** Eight datasets on host protein interactions with the SARS-CoV-2 RNA.

**Datasets**	**Number**	**Methods**	**Strain**	**GISAID**	**Ref**
Dataset 1	332	cRIC; RAP-MS	England/02/2020	EPI_ISL_407073	[22]
Dataset 2	130	ChIRP-M/S	France/IDF-220-95/2020	EPI_ISL_469284	[23]
Dataset 3	65	MS	IPBES-YL01/2020	–	[24]
Dataset 4	206	RAP-MS; LC–MS/MS	South Korea/KCDC/2020	EPI_ISL_407193	[25]
Dataset 5	181	RAP–MS; LC–MS/MS	Not specified	–	[14]
Dataset 6	142	ChIRP-M/S	IPBCAMS-YL01/2020	–	[26]
Dataset 7	257	RaPID; LC–MS/MS	Wuhan-Hu-1/2019	EPI_ISL_402125	[27]
Dataset 8	278	ChIRP-M/S	USA/WA1/2020	EPI_ISL_404895	[28]

### Functional enrichment analysis of host proteins interacting with SARS-CoV-2 RNA

Functional enrichment analysis were performed on the eight datasets using the ClusterProfiler software (version 4.4.4) [1]. This analysis focused on three key areas: KEGG pathways, the Reactome database, and GO. The results were generated using the plotting package in the R environment (version 4.2.1).

### Comprehensive identification and network analysis of cross-species RNA virus-host protein interactions

To explore common interaction mechanisms, we performed a comparative analysis against IAV, ZIKV, and DENV. Host protein interactome data for these non-SARS-CoV-2 RNA viruses were retrieved from *Rvvictor*, our curated database of RNA virus RNA—host protein interactions [29], which archives 16,223 experimentally supported interactions across 107 virus/strains. We selected these viruses based on three criteria: (i) they are pathogenic ssRNA viruses dependent on host cytoplasmic translation machinery, ensuring biological comparability with SARS-CoV-2; (ii) their interactome data were generated by experimentally validated methods (*e.g.* RAP-MS, ChIRP-MS) comparable to those used for SARS-CoV-2; and (iii) their datasets contained sufficient host proteins for robust cross-viral enrichment analysis. Among all viruses in *Rvvictor*, only IAV (1545 host proteins), DENV (664 proteins), and ZIKV (530 proteins) met all three criteria and were therefore included in this analysis. By comparing these with the host proteins interacting with SARS-CoV-2, key host proteins common to all four viruses were identified and selected. Subsequently, functional annotation analysis was performed using the DAVID tool, and the interaction network was visualized with Cytoscape software.

### Drug repurposing strategy for host protein targets in cross-virus interactions

To identify candidate drugs targeting host proteins involved in cross-virus interactions, we performed a structured, literature-based pharmacological annotation. For each of the 275 conserved host proteins, we used keyword combinations such as ‘host protein name + drug’ and ‘SARS-CoV-2 + drug repurposing’ to search for relevant studies. Drug–protein pairs were retained if the host protein was reported with direct experimental evidence as a molecular target of the compound (*e.g.* binding, inhibition or functional modulation in biochemical) and the study provided sufficient information to unambiguously map both the protein and the compound. For each retained pair, we recorded the drug name, PubMed ID and, when available, the DrugBank identifier. This procedure identified 35 drugs targeting 21 conserved host proteins involved in interactions with IAV, ZIKV, DENV and SARS-CoV-2 RNA.

### Protein–protein interaction analysis

Protein–protein interaction (PPI) analysis was conducted using the STRING database (https://string-db.org/) to investigate interactions among the 275 shared host proteins, to identify critical interaction points in the viral infection and replication process. A confidence threshold of >0.9 was selected to ensure the high accuracy and reliability of the results, thus accurately pinpointing interactions that have a decisive impact on the viral life cycle.

## Results and discussions

### KEGG pathway analysis identifies host factors associated with SARS-CoV-2 viral replication and immune regulation

**Figure 2:**
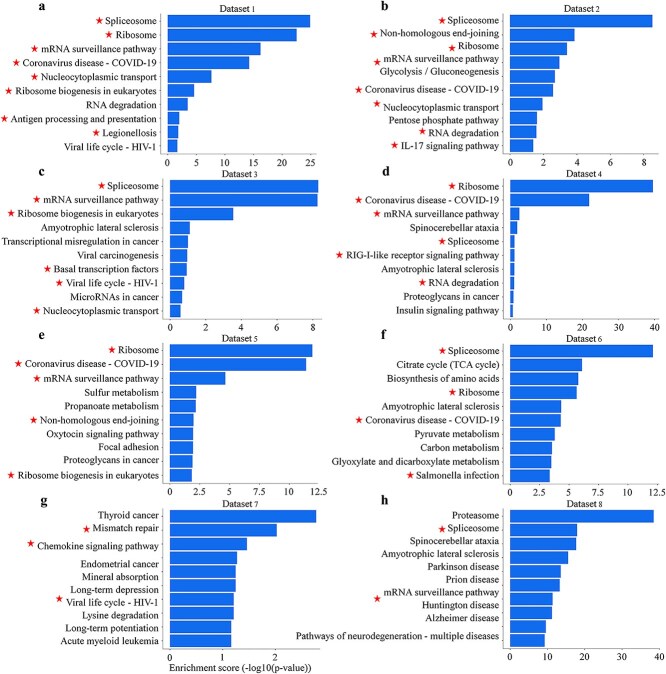
Bar plots show the top 10 significantly enriched KEGG pathways for each of the eight independent datasets of SARS-CoV-2 RNA–interacting host proteins. The x-axis indicates the enrichment score (−log10(adjusted P-value)). Only pathways with adjusted *P*-value <0.05 are shown, and for each dataset the top 10 pathways are displayed. Stars (★) mark key RNA-processing and translation-related pathways that recur across multiple datasets (such as Spliceosome, Ribosome, and mRNA surveillance pathway).

Previous transcriptomic and proteomic studies have shown that SARS-CoV-2 infection affects host RNA metabolism, translation, and innate immune pathways, which are thought to support viral replication and help the virus cope with antiviral defenses [30]. Consistent with these reports, our KEGG pathway enrichment analysis of host proteins interacting with the SARS-CoV-2 RNA genome revealed significant enrichment (adjusted P-value <0.05) of pathways related to RNA biology and protein synthesis (Fig. 2a–h). The most frequent terms across the eight datasets were ‘Ribosome’, ‘Spliceosome’, ‘mRNA surveillance pathway’, and ‘RNA degradation’. Together, these results suggest that SARS-CoV-2 RNA is closely associated with proteins involved in mRNA processing, quality control, and translation.

We also observed repeated enrichment of infection- and immune-related pathways, such as ‘viral life cycle – HIV-1’, ‘Coronavirus disease – COVID-19’, ‘IL-17 signaling’, ‘RIG-I-like receptor signaling’, and ‘chemokine signaling’. The red-starred pathways (*e.g.* ‘Spliceosome’, ‘Ribosome’, ‘mRNA surveillance pathway’, ‘Coronavirus disease – COVID-19’) appear in several datasets, which suggests that similar groups of host factors are recovered even under different experimental conditions.

In addition, some of the enriched terms were related to neurodegenerative disease (e.g. ‘amyotrophic lateral sclerosis’, ‘Parkinson disease’, ‘Huntington disease’) and cancer biology (e.g. ‘transcriptional misregulation in cancer’, ‘viral carcinogenesis’). Rather than implying direct disease comorbidity, these enrichments highlight the pleiotropic nature of core RNA-binding and translation-related proteins, which are conserved across tissues and frequently dysregulated in both viral infection and these pathological conditions. We also noted enrichment of metabolic pathways such as ‘glycolysis/gluconeogenesis’, ‘pentose phosphate pathway’, ‘citrate cycle (TCA)’, and ‘pyruvate metabolism’, which may point to links between viral RNA–binding proteins and the rewiring of central carbon metabolism.

Taken together, these KEGG results suggest that SARS-CoV-2 RNA interacts with a conserved group of host factors involved in mRNA processing, translation, immune and infection-related signaling, and metabolism, placing the viral genome within core cellular systems that control gene expression and antiviral defense.

### Reactome pathway analysis highlights the enrichment of host translation machinery in the SARS-CoV-2 interactome

To delve deeper into the specific molecular mechanisms driving these interactions, we utilized Reactome pathway enrichment analysis (Supplementary Fig. 1A–H). While the KEGG analysis highlighted broad functional categories, the Reactome results provided granular insight into the specific steps of protein synthesis targeted by the virus. Consistent with the viral requirement for host ribosomes [31–33], we found a significant and repeated enrichment of terms governing ‘Translation initiation complex formation’, ‘Cap-dependent translation initiation’, and ‘Ribosomal scanning.’ Specifically, the enrichment of terms such as ‘Formation of a pool of free 40S subunits’ and ‘Activation of the mRNA upon binding of the cap-binding complex’ suggests that SARS-CoV-2 RNA actively recruits the rate-limiting components of the host translation initiation machinery to ensure the efficient synthesis of viral proteins.

Beyond translation initiation, the analysis revealed a strong association with RNA quality control and processing mechanisms. In several datasets (*e.g.* Datasets 2, 4, and 6), we observed high enrichment for ‘mRNA Splicing’ and ‘Nonsense-Mediated Decay (NMD).’ As NMD is a host surveillance mechanism capable of degrading viral RNA, the interaction with NMD factors may reflect the virus’s strategy to inhibit this pathway and protect its genome [34]. Furthermore, we detected a specific enrichment of the ‘L13a-mediated translational silencing of Ceruloplasmin expression’ pathway. L13a is a key component of the GAIT (Gamma-interferon-Activated Inhibitor of Translation) complex, which selectively silences inflammatory transcripts. This suggests that SARS-CoV-2 may exploit this mechanism to modulate the host immune response by dampening the translation of specific inflammatory genes. Finally, the identification of signaling pathways, such as FGFR2 signaling, in multiple datasets indicates a possible intersection between viral RNA–protein interactions and host receptor-mediated signaling cascades.

### Enrichment of host RNA processing, metabolic and neurodegeneration-related pathways among SARS-CoV-2 RNA-interacting proteins

To elucidate the broader signaling and metabolic networks engaged by SARS-CoV-2 RNA, we performed WikiPathways enrichment analysis using the host proteins that interacted with the viral RNA genome (Supplementary Fig. 2A–H). Like our KEGG and Reactome results, the most significantly enriched terms included ‘mRNA processing’, ‘Translation factors’, and ‘Cytoplasmic ribosomal proteins’, as well as the SARS-CoV-2-specific pathway ‘nsp1 from SARS-CoV-2 inhibits translation initiation.’ These findings support the view that SARS-CoV-2 targets host RNA processing and translation machinery, in line with experimental evidence that viral nsp1 and related factors suppress host protein synthesis while favoring viral mRNA translation [35, 36].

Beyond protein synthesis, several enriched pathways pointed to altered cell signaling and central carbon metabolism, including ‘VEGFA–VEGFR2 signaling pathway’, ‘TCA cycle’, and ‘Cholesterol metabolism’. These results are consistent with reports that SARS-CoV-2 infection reprograms host energetics and exploits VEGF-related signaling to support viral replication and modulate inflammatory responses [37, 38].

We also observed a distinct enrichment of innate immune defense pathways, such as ‘Cellular components of RIG-I-like receptor pathway’ and ‘Perturbations to host-cell autophagy’. This suggests that viral RNA–binding proteins directly intersect with cellular surveillance systems, potentially to evade innate RNA sensing and autophagy-linked antiviral defenses [39, 40].

Finally, our WikiPathways analysis highlighted terms associated with neurodegeneration and protein homeostasis, including ‘Alzheimer’s disease and miRNA effects’, ‘Parkin-ubiquitin proteasomal system pathway’, and ‘Alzheimer’s disease’. These enrichments align with recent studies reporting genetic and molecular links between COVID-19 and neurodegenerative disorders [41, 42]. Crucially, these findings raise the possibility that SARS-CoV-2 affects neuronal homeostasis not just through direct infection, but through systemic stress on RNA regulation and protein degradation machinery. Together, these results complement our KEGG and Reactome analyses by showing that SARS-CoV-2 RNA–interacting proteins are embedded in key metabolic, immune, and neuro-molecular networks.

### GO analysis reveals association of SARS-CoV-2 RNA with host mRNA processing, translation, and splicing machinery, implicating support for viral replication

**Figure 3:**
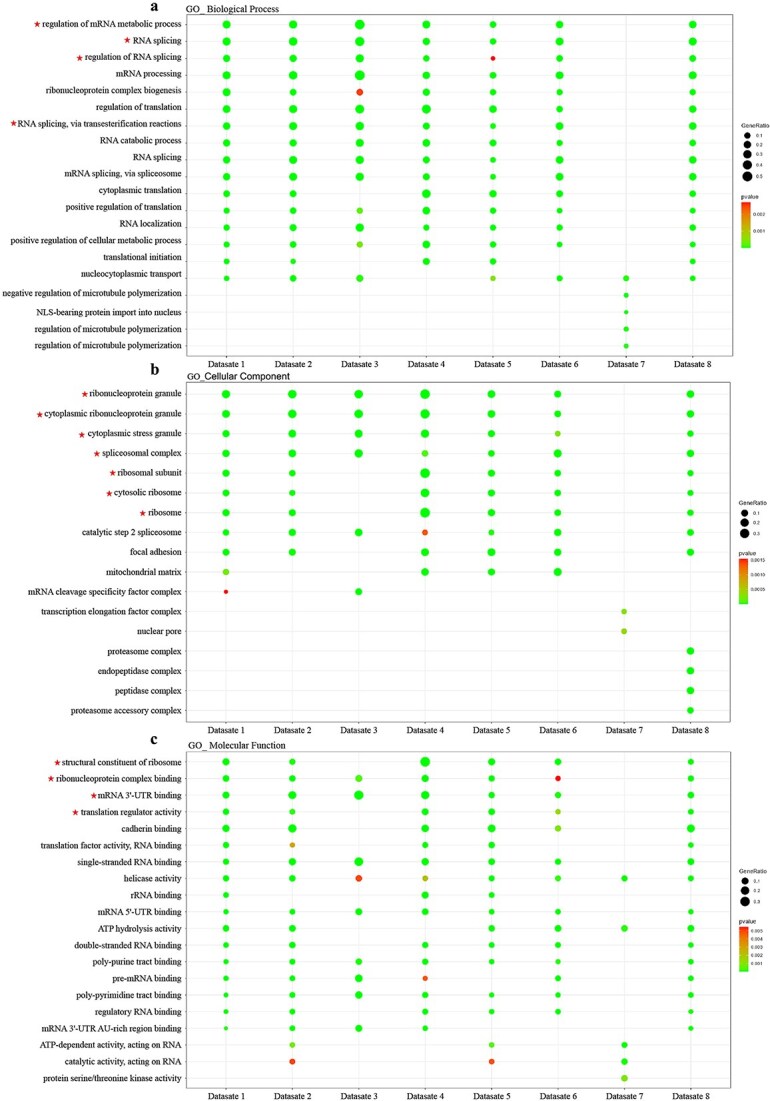
GO enrichment of host proteins that interact with the SARS-CoV-2 RNA genome across eight datasets. (a–c) Dot plots show significantly enriched GO terms for host proteins interacting with the SARS-CoV-2 RNA genome in each of the eight datasets, separated into (a) Biological Process (BP), (b) Cellular Component (CC), and (c) Molecular Function (MF) categories. The size of each dot represents the gene ratio, and the color scale indicates the adjusted P-value. Only terms with adjusted P-value <0.05 are shown (up to the top 20 terms per dataset). Red stars (★) highlight key RNA-processing, splicing, and translation-related terms that recur across multiple.

To define the specific cellular machinery engaged by SARS-CoV-2 RNA, we performed GO enrichment analysis (Fig. 3a–c). In terms of Biological Processes, the viral RNA interactome was dominated by terms related to post-transcriptional regulation, specifically ‘RNA splicing’, ‘regulation of mRNA metabolic process’, and ‘mRNA processing’ (Fig. 3a). This indicates that beyond simple translation, viral RNA–associated proteins are heavily involved in the maturation and remodeling of the host transcriptome.

Cellular Component analysis revealed that these interactions occur within specific ribonucleoprotein assemblies (Fig. 3b). While we observed expected enrichment for ‘ribosomal subunit’ and ‘cytosolic ribosome’, we also found significant enrichment for the ‘spliceosomal complex’ and ‘cytoplasmic stress granules’. This suggests that SARS-CoV-2 RNA actively localizes to key processing centers involved in both RNA splicing and the cellular stress response.

Finally, regarding molecular function, the interactome was enriched for ‘RNA binding’, ‘mRNA 3’-UTR binding’, and ‘structural constituent of ribosome’ (Fig. 3c). Collectively, these GO results suggest that SARS-CoV-2 associates with a specialized set of host machinery potentially involved in mRNA processing, translation efficiency, and stress granule modulation.

### Cross virus analysis identifies shared host factors and potential antiviral targets

**Figure 4:**
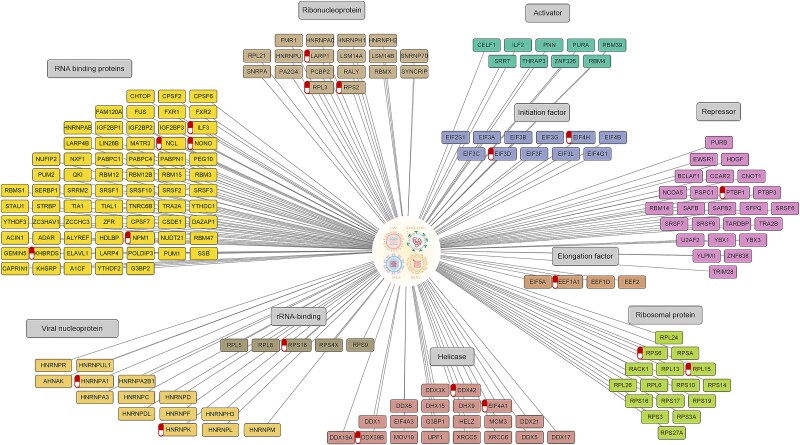
Comprehensive analysis and network visualization of Shared host proteins targeted by four RNA viruses and their functional categories. Network visualization of 275 host proteins that interact with the RNA genomes of IAV, ZIKV, DENV, and SARS-CoV-2. Nodes are grouped and colored by functional category, including ‘RNA-binding proteins’, ‘ribonucleoproteins’, ‘viral nucleoproteins’, ‘ribosomal proteins’, ‘helicases’, ‘translation initiation factors’, ‘elongation factors’, ‘activator factors’, ‘repressor factors’, and ‘rRNA-binding proteins.’ Red drug symbols mark host proteins with at least one known approved or experimental small-molecule drug. In total, 35 drugs targeting 21 shared host proteins were identified.

This study aimed to elucidate conserved host dependencies shared across infections by four RNA viruses: IAV, Zika virus, DENV, and SARS-CoV-2. We first retrieved host protein interactome data for IAV, Zika, and DENV from our developed *Rvvictor* database, identifying 1545, 530, and 664 host proteins, respectively. These datasets were then compared with the host proteins interacting with SARS-CoV-2 RNA (Supplementary Table 1). Through this integrative analysis, we identified 275 host proteins that interact with all four viral RNAs, suggesting a core set of shared host factors that are broadly exploited during RNA virus infection (Fig. 4).

To explore the functional implications of these common host factors, we performed functional annotation using the DAVID tool and visualized the results in Cytoscape (Fig. 4). Proteins were grouped into major functional categories, most notably translational machinery (ribosomal proteins, initiation and elongation factors), RNA processing complexes (helicases, ribonucleoproteins), and regulatory factors. To assess therapeutic potential, we mapped known small-molecule modulators to this network. In total, we identified 35 candidate drugs targeting 21 of the shared host proteins, providing a preliminary resource for prioritizing potential broad-spectrum antiviral targets.

### Shared host protein networks across RNA viruses provide insights into broad-spectrum antiviral targets

**Figure 5:**
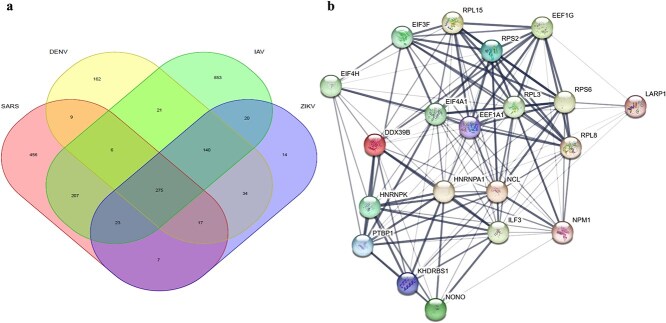
Conserved host protein interactions and functional network analysis across four RNA viruses. (a) Venn diagram depicting the interactome overlap between SARS-CoV-2, DENV, IAV, and ZIKV. The intersection identifies 275 conserved host proteins shared across all four viral infections. (b) PPI network of the 275 shared proteins (STRING database; confidence >0.9). The network highlights key translational machinery, including translation factors (*e.g.* EEF1A1, EIF4A1) and ribosomal proteins (*e.g.* RPL3, RPS6). Nodes involved in RNA processing and stability (*e.g.* NCL, ILF3) and immune regulation are also marked. Promising antiviral drug targets, such as EEF1A1 and NCL, are emphasized to illustrate potential avenues for broad-spectrum therapeutic intervention.

To map the functional connectivity of the 275 shared host proteins, we performed a PPI analysis using the STRING database. Focusing on high-confidence interactions (score > 0.9), we constructed a core interaction network (Fig. 5a, b). The results revealed critical roles of host proteins in the translation of viral RNA and protein synthesis, with a particular enrichment of translation factors (*e.g.* EEF1A1, EIF4A1, EIF4H, EIF3H) and ribosomal proteins (*e.g.* RPL3, RPS6, RPL8, RPL15, RPS2). These proteins are known to facilitate efficient translation, raising the possibility that their interaction with viral components may support viral replication. Notably, EEF1A1 has previously been highlighted for its essential role in SARS-CoV-2 replication [43].

Additionally, proteins such as Nucleolin (NCL), ILF3, NPM1, NONO, PTBP1, HNRNPK, and DDX39B were identified as pivotal players in RNA processing, including splicing, nuclear export, transport, and stability. This suggests that the virus may manipulate these proteins to disrupt host RNA metabolism and facilitate its own replication [44]. In the context of immune regulation, proteins like ILF3 and NCL are of particular interest as they participate in interferon signaling pathways critical for combating viral infections [45].

The recurrent reliance on these host proteins by distinct RNA viruses highlights a conserved dependency, offering new perspectives for broad-spectrum antiviral design. Specifically, EEF1A1, NCL, EIF4A1, and ILF3 emerged as key functional hubs and potential drug targets. For instance, small-molecule inhibitors targeting EEF1A1 may effectively block viral protein translation [46]. Similarly, therapeutic strategies targeting NCL have shown promise in blocking viral interactions [47], while the modulation of EIF4A1 and ILF3 represents a potential avenue for antiviral treatment [48, 49]. These findings provide a theoretical foundation for understanding how viruses exploit host mechanisms and offer valuable directions for developing targeted antiviral strategies.

To validate the functional significance of our consensus interactome, we compared our findings with genome-wide CRISPR-Cas9 screens that identify host factors essential for SARS-CoV-2 replication. Notably, Flynn *et al.* demonstrated that the SARS-CoV-2 RNA interactome is functionally enriched for both proviral and antiviral factors [28]. Consistent with our network analysis, translation factors and RNA-binding proteins such as EEF1A1 and CNBP were identified as functional proviral dependencies, where their depletion significantly reduced viral replication or rescued cell death. Conversely, shared host factors such as NONO and HNRNPs were identified as key antiviral RNA sensors that bind viral RNA to restrict replication. This convergence of our meta-analysis with functional genetic screening data confirms that these conserved interactions represent critical bottlenecks in the viral life cycle and viable targets for therapeutic intervention.

## Conclusion

In this study, we integrated eight published SARS-CoV-2 RNA–protein interactomes and compared them with the viral RNA interactomes of IAV, ZIKV, and DENV to define conserved virus—host interactions in RNA virus infection. Across independent datasets and analysis frameworks (KEGG, Reactome, WikiPathways, and GO), functional enrichment and network analyses repeatedly highlighted pathways involved in mRNA processing, translation, RNA surveillance and innate immune regulation as central host processes engaged by viral RNAs.

By intersecting the four viral RNA interactomes, we identified a core set of 275 host proteins that interact with all four RNA viruses and form highly connected protein–protein interaction modules. These shared factors include translation and initiation components, ribosomal proteins, and RNA-binding proteins such as EEF1A1, EIF4A1, NCL, and ILF3, which together are positioned to influence viral RNA replication, localization and protein synthesis. This cross-virus perspective shifts the focus from virus-specific interactions to conserved host dependencies, providing a conceptual basis for host-directed, potentially broad-spectrum antiviral strategies.

At a translational level, annotating the 275 shared proteins with existing drug–target information allowed us to prioritize 21 host factors with 35 small-molecule modulators as candidate nodes for host-directed antiviral repurposing. These candidates, together with the conserved interaction modules described here, provide a focused shortlist for subsequent experimental testing.

Despite these insights, our study has several limitations. It is based entirely on previously published interactome datasets, which may be influenced by differences in cell type, experimental platform and viral strain. In addition, the drug–target layer is derived from literature and database curation rather than newly generated functional data. The pathways, proteins and drug–target pairs we highlight should therefore be regarded as hypothesis-generating resources, not as validated therapeutic strategies. Future work should experimentally test the roles of the prioritized host factors in viral replication and immune control, evaluate the effects of their pharmacological modulation in relevant infection models, and expand this integrative framework to additional viruses and patient-derived datasets. In summary, our findings provide a systems-level view of how diverse RNA viruses converge on shared host networks and offer a starting point for the rational design of host-directed antiviral interventions.

## Supplementary Material

Amahong_et_al_SI_17-dec-2025_revised_elag001

Sup_Table_1_elag001

## Data Availability

The datasets used in this study are provided in Supplementary Table 1.

## Notes

**Kuerbannisha Amahong** is a Postdoctoral Researcher at the Institute of Biomedicine, University of Gothenburg. Her research focuses on the molecular mechanisms of RNA-binding proteins and virus-host interactions.

**Yuhong Liu** is a Master’s student at the College of Pharmaceutical Sciences, Hangzhou Normal University. Her research interests focus on RNA viruses and the complex dynamics of virus-host interactions.

**Zheng Zhang** is a Bachelor's student at the Department of Medicine, Hangzhou Normal University. His research interests lie in bioinformatics and the computational analysis of virus-host interactions.

**Lin Tao** is a Distinguished Professor at Hangzhou Normal University. His research focuses on computer-aided drug design (CADD) and the application of machine learning in pharmaceutical discovery.

**Aishe Sarshad** is an Associate Professor at the Institute of Biomedicine, University Gothenburg. Her research investigates RNA-binding proteins and RNA interference to uncover gene regulation mechanisms and target disease-driving genes.

**Feng Zhu** is a Tenured Professor at the College of Pharmaceutical Sciences, Zhejiang University. His research integrates medicinal chemistry, bioinformatics, and systems biology to advance drug design and target discovery.
