# Supplementary material for: An integrative meta-analysis of SARS-CoV-2 RNA–protein interactomes identifies conserved host factors shared with other RNA viruses
Source: Brief Funct Genomics. 2026 May 18;25:elag001. doi: 10.1093/bfgp/elag001 (PMC13182571; doi:10.1093/bfgp/elag001)
Supplement: Amahong_et_al_SI_17-dec-2025_revised_elag001 [file amahong_et_al_si_17-dec-2025_revised_elag001.docx]

**Supplemental Material**

**An Integrative Meta-analysis of SARS-CoV-2 RNA–Protein Interactomes Identifies Conserved Host Factors Shared with Other RNA Viruses**

Kuerbannisha Amahong^1,2,3^, Yuhong Liu ^4^, Zheng Zhang^4^, Lin Tao^4^, Aishe A. Sarshad^2,3^* and Feng Zhu^1^*

**Supplemental Figures**


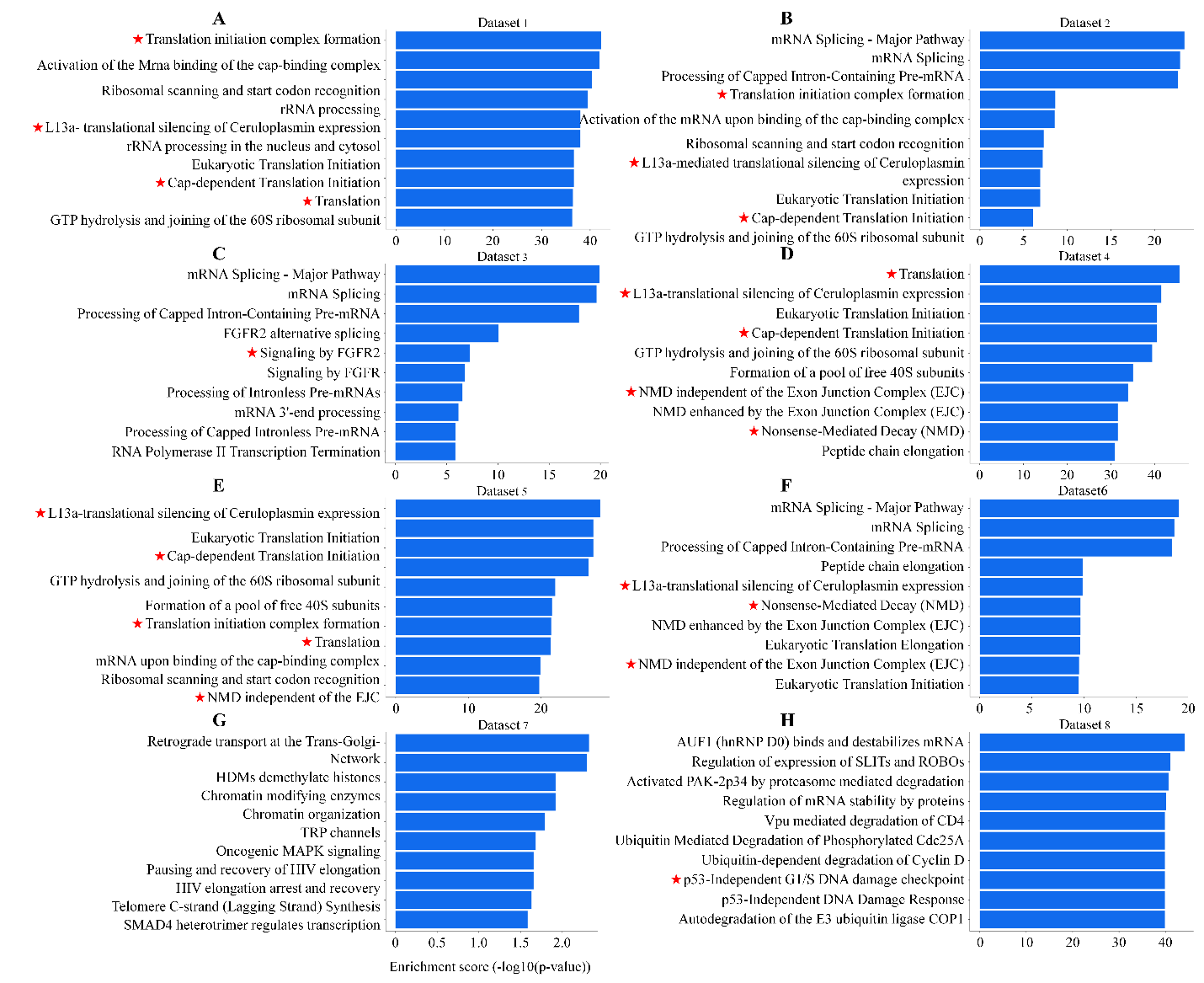


**Supplemental Figure 1.** Bar plots show the top ten significantly enriched Reactome pathways for each of the eight independent datasets of SARS-CoV-2 RNA–interacting host proteins. The x-axis indicates the enrichment score (−log10(adjusted P-value)). Only pathways with adjusted P-value < 0.05 are shown, and for each dataset the top 10 pathways are displayed. Stars (★) mark key RNA-processing and translation-related pathways that recur across multiple datasets.

**Alt text (Supplemental Figure 1):** Eight bar plots showing Reactome pathway enrichment across eight SARS-CoV-2 RNA interactome datasets; stars mark recurring RNA-processing and translation pathways.


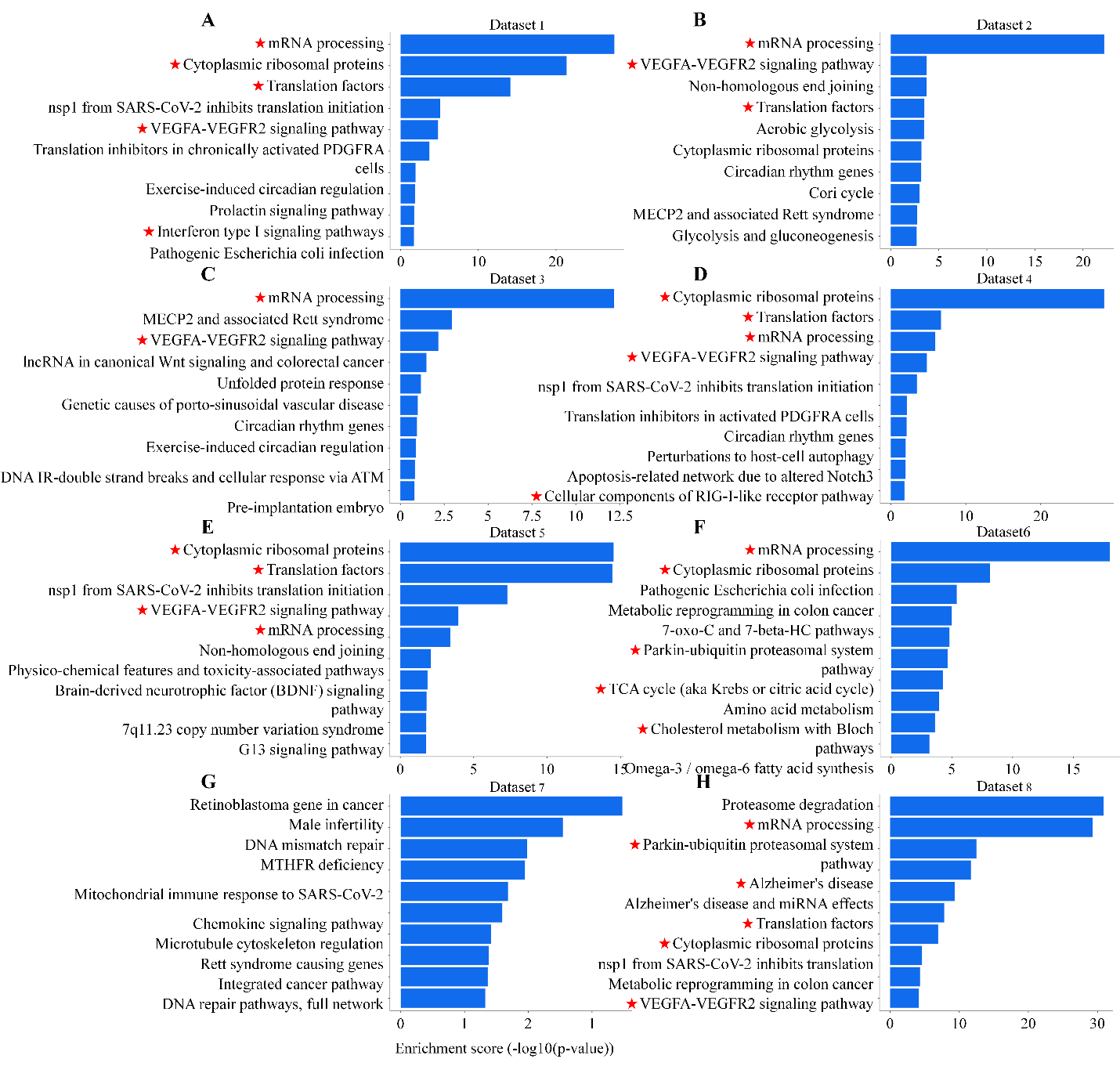


**Supplemental Figure 2**. Bar plots show the top ten significantly enriched WikiPathways for each of the eight independent datasets of SARS-CoV-2 RNA–interacting host proteins. The x-axis indicates the enrichment score (−log10(adjusted P-value)). Only pathways with adjusted P-value < 0.05 are shown, and for each dataset the top 10 pathways are displayed. Stars (★) mark key RNA-processing and translation-related pathways that recur across multiple datasets

**Alt text (Supplemental Figure 2):** Eight bar plots showing WikiPathways enrichment across eight SARS-CoV-2 RNA interactome datasets; stars mark recurring RNA-processing and translation pathways.
